# Similar patterns of rDNA evolution in synthetic and recently formed natural populations of *Tragopogon *(Asteraceae) allotetraploids

**DOI:** 10.1186/1471-2148-10-291

**Published:** 2010-09-22

**Authors:** Hana Malinska, Jennifer A Tate, Roman Matyasek, Andrew R Leitch, Douglas E Soltis, Pamela S Soltis, Ales Kovarik

**Affiliations:** 1 Institute of Biophysics, Academy of Sciences of the Czech Republic, v.v.i, Laboratory of Molecular Epigenetics, Kralovopolska 135, CZ-61265 Brno, Czech Republic; 2 Institute of Molecular BioSciences, Massey University, Palmerston North 4442, New Zealand; 3 School of Biological Sciences, Queen Mary University of London, E1 4NS, UK; 4 Department of Biology, University of Florida, Gainesville, FL 32611, USA; 5 Florida Museum of Natural History, University of Florida, Gainesville, FL 32611, USA

## Abstract

**Background:**

*Tragopogon mirus *and *T. miscellus *are allotetraploids (2*n *= 24) that formed repeatedly during the past 80 years in eastern Washington and adjacent Idaho (USA) following the introduction of the diploids *T. dubius*, *T. porrifolius*, and *T. pratensis *(2*n *= 12) from Europe. In most natural populations of *T. mirus *and *T. miscellus*, there are far fewer 35S rRNA genes (rDNA) of *T. dubius *than there are of the other diploid parent (*T. porrifolius *or *T. pratensis*). We studied the inheritance of parental rDNA loci in allotetraploids resynthesized from diploid accessions. We investigate the dynamics and directionality of these rDNA losses, as well as the contribution of gene copy number variation in the parental diploids to rDNA variation in the derived tetraploids.

**Results:**

Using Southern blot hybridization and fluorescent *in situ *hybridization (FISH), we analyzed copy numbers and distribution of these highly reiterated genes in seven lines of synthetic *T. mirus *(110 individuals) and four lines of synthetic *T. miscellus *(71 individuals). Variation among diploid parents accounted for most of the observed gene imbalances detected in F_1 _hybrids but cannot explain frequent deviations from repeat additivity seen in the allotetraploid lines. Polyploid lineages involving the same diploid parents differed in rDNA genotype, indicating that conditions immediately following genome doubling are crucial for rDNA changes. About 19% of the resynthesized allotetraploid individuals had equal rDNA contributions from the diploid parents, 74% were skewed towards either *T. porrifolius *or *T. pratensis*-type units, and only 7% had more rDNA copies of *T. dubius*-origin compared to the other two parents. Similar genotype frequencies were observed among natural populations. Despite directional reduction of units, the additivity of 35S rDNA locus number is maintained in 82% of the synthetic lines and in all natural allotetraploids.

**Conclusions:**

Uniparental reductions of homeologous rRNA gene copies occurred in both synthetic and natural populations of *Tragopogon *allopolyploids. The extent of these rDNA changes was generally higher in natural populations than in the synthetic lines. We hypothesize that locus-specific and chromosomal changes in early generations of allopolyploids may influence patterns of rDNA evolution in later generations.

## Background

Chromosome counts suggest that between 30 and 100% of angiosperm species are polyploids [1], and Wood et al. [2] propose that 15% of angiosperm speciation events are associated with polyploidy whereas recent genomic studies of selected model and crop species have revealed that all plant genomes sequenced to date have signatures of one or more whole-genome duplications in their evolutionary history [3,4]. The success of newly formed angiosperm polyploids is partly attributable to their highly plastic genome structure as manifested by deviations from Mendelian inheritance of genetic loci and chromosome aberrations [5]. Indeed, there are numerous examples of intergenomic exchanges, chromosomal translocations, transposon proliferation, and sequence loss in both newly formed and ancient allopolyploid species (for review see [6,7]).

In plants, nuclear ribosomal DNA (rDNA) units occur in tandem arrays at one or several loci (for review see [8,9]). Each large 35S rDNA unit contains the 18S, 5.8S, and 26S rRNA genes, the internal transcribed spacers (ITS), and the intergenic spacer (IGS). The 5S genes encoding 120-nt transcripts are usually, but not always [10], located at different chromosomal loci than 35S rDNA. The genes are highly conserved even between eukaryotes and prokaryotes, whereas divergence of ITS is sufficient to resolve species relationships within most genera [11]. The IGS, which contains the transcription start site and genetic and epigenetic features that influence the regulation of the downstream genes, diverges rapidly, and substantial differences in structure may occur even within a species [12-14]. The number of gene copies may vary from 500 up to tens of thousands in certain plant species [15]. Similar variation has been observed in locus number, with levels ranging from one to several loci per haploid set [16]. Within species, the copy and locus number is usually stable, although intraindividual and intergenerational variation in copy number has been reported in some plants [17]. As with other repeated sequences, rDNA can undergo concerted evolution involving sequence homogenization [18,19]. Such a process efficiently eliminates mutated copies maintaining long arrays of functional tandemly arranged genes.

The behavior of rDNA in allopolyploids has attracted considerable attention because it is used as a molecular and cytogenetic marker of allopolyploidy [20]. Indeed, the hybrid origin of many species has been successfully deciphered using ITS sequences. Nevertheless, repeat and locus loss, and intra- and interlocus recombination seem to be ongoing evolutionary processes, potentially preventing identification of hybrids. In fact, many well-defined allopolyploid species have either lost one or several loci [21-23], eliminated or contracted parental arrays [24-26], recombined [27] or replaced the units [28,29]. On the other hand, some polyploid species seem to maintain both parental copies in the genome long after allopolyploid formation [14,30-33]. These studies indicate that the process of rDNA evolution is complicated, and that no firm conclusion can be drawn on the tempo and direction of repeat homogenization. Nevertheless, there are examples of synthetic allopolyploid lines in which rDNAs have already undergone rearrangements at the chromosomal and unit levels [13,34,35].

Recently formed allopolyploids represent unique natural systems in which to study the immediate consequences of allopolyploidy. Only a few polyploid plant species are known to have formed in the past 200 years: *Cardamine schulzii *[36], *Spartina anglica *[37], *Senecio cambrensis *and *S. eboracensis *[38], *Tragopogon mirus *and *T. miscellus *[39]. *Cardamine *allopolyploid populations seem to evolve recombined ITS types [40]. In *Spartina anglica*, the two individuals collected from different localities differed in the composition of rDNA units [41].

Allotetraploid *Tragopogon mirus *and *T. miscellus *formed in the Palouse region (eastern Washington, and western Idaho, USA) within the last 80 years [39,42] and thus represent an excellent model for examining early events in allopolyploid evolution. Recent studies using different methodological approaches have shown frequent loss of homeologous sequences, including low-copy protein-coding genes [43,44] and high-copy rDNA [45]. In the latter, a population-level analysis revealed reduction of rDNA arrays derived from the *T. dubius *diploid parent in all but one natural population examined (*T. mirus*). While the average magnitude of gene loss was about 50%, there were individuals that lost as many as 95% of all repeats. Cytogenetic studies confirmed that rearrangements were homologous and were not linked to chromosome loss [46].

The interpretation of genetic variation in natural polyploids is always complicated by the fact that genetic parents are unknown even in the case of recently formed species. That is, some allopolyploids may start out with far more rDNA copies of one parent than the other, simply because the diploid parents differ in copy number. On the other hand, genotypic variation may arise from genetic changes induced by stressful conditions experienced during allopolyploidy [5]. In this study we asked: (i) What is the contribution of parental diploids to copy number variation in newly formed allopolyploid *T. mirus *and *T. miscellus*? (ii) What are the dynamics of rDNA rearrangements, and do they occur suddenly or gradually? (iii) Is there a directionality and genetic predisposition for locus rearrangement? (iv) Are there parallels in the evolution of rDNA in natural and synthetic populations of the two allopolyploids? (v) What are the likely mechanisms of rDNA rearrangement? To address these questions we synthesized allotetraploid lines of *T. mirus *and *T. miscellus *[47] using several different populations of parental accessions. We determined homeolog gene ratios by Southern blot and slot blot hybridization. The locus numbers were assessed using FISH. Evidence was obtained for repeat loss in early generations of synthetic allopolyploid lines at frequencies and directionality similar to those observed in natural situations.

## Methods

### Plant material

Field-collected seeds of three diploid *Tragopogon *species were planted and selfed for one generation. Then 103 different crosses were made between individuals from three populations of *T. dubius *(2613, 2615, 2616), two populations of *T. porrifolius *(2607, 2611), and two populations of *T. pratensis *(2608, 2609) from different localities (Table 1, Table 2 and [47]). Seeds from successful crosses were treated with 0.1 or 0.25% (w/v, water solution) colchicine during germination (overnight), washed, and placed in pots with soil. Approximately 6 months after germination, young plants were genotyped to determine their parentage using a marker (TDF 85) specific for all three diploid species [43,47]. The non-treated F_1 _diploid hybrids were planted as controls. Detailed information about crosses is described in [44,47].

**Table 1:** Parental origin of synthetic allotetraploids and direction of crosses

	*T. mirus*							*T. miscellus*			
	
Line	70	73	98	116	121	134	135	67	79	111	129
**parents** ♀ × ♂	2611 × 2613	2611 × 2613	2611 × 2613	2607 × 2615	2615 × 2607	2607 × 2613	2613 × 2607	2609 × 2616	2609 × 2616	2608 × 2613	2613 × 2608
^1^N	9	48	7	28	8	14	16	15	22	39	3
^2^S_0_	2	3	1	1	0	1	1	2	1	4	1
^2^S_1_	7	34	6	27	8	13	15	13	21	35	2
^2^S_2_	0	11	0	0	0	0	0	0	0	0	0

^1^total number of individuals analyzed by Southern blot hybridization.

^2^numbers in S_0_-S_2 _generations; the S_2 _was the progeny of selfed S_1 _parents (73-14, 73-1, 73-2) that have been analyzed in this study by molecular and cytogenetic approaches.

**Table 2:** Characteristics of rDNA loci in populations of parental diploid species used for construction of synthetic lines

*Species*	***^1^Collection no***.	*Location*	*^2^rDNA copies*	*rDNA* *genotype*	*No. of 35S rDNA sites*	*No. of 5S rDNA sites*
*T. porrifolius*	2607	Troy, ID	1.5 ± 0.3	High-copy	4	4
	2611	Pullman, WA	1.0 ± 0.2	Low-copy	4	4
*T. pratensis*	2608	Moscow, ID	1.2 ± 0.1	Medium-copy	2	2
	2609	Spangle, WA	1.5 ± 0.2	High-copy	2	2
*T. dubius*	2613	Pullman, WA	1.7 ± 0.4	High-copy	2	2
	2615	Spokane, WA	0.9 ± 0.1	Low-copy	2	2
	2616	Spangle, WA	1.0 ± 0.2	Low-copy	2	2

^1^Soltis & Soltis collection numbers; vouchers deposited at FLAS.

^2^DNA copies per haploid set in thousands.

### Molecular cytogenetic analysis

Root tips cut from vigorously growing plants were pre-treated with 2 mM 8-hydroxyquinoline (Sigma-Aldrich Company Ltd, Poole, Dorset, UK) to obtain metaphase nuclei. After 2 hours of incubation on ice, root tips were fixed in ethanol: acetic acid (3: 1) at room temperature overnight, then stored in 70% ethanol at -20°C. Fixed root tips were digested in 0.3% (w/v) cellulase Onozuka R-10 (Apollo Scientific Ltd, Stockport, Cheshire, UK), 0.3% (w/v) pectolyase Y23 (MP Biomedicals, Solon, Ohio, USA), and 0.3% (w/v) drieselase (Sigma-Aldrich Company Ltd., Poole, Dorset, UK) for 27 min and transferred to 1% citrate buffer pH 4.8 and incubated for 1 hour. The meristematic cells behind the root cap were squashed onto a glass slide in a drop of 60% acetic acid. Coverslips were removed after freezing in liquid nitrogen.

Fluorescent *in situ *hybridization (FISH) of the diploids and polyploids followed standard protocols [48]. The probe for 5S rDNA was prepared by PCR amplification of the cloned *Nicotiana tabacum *5S rRNA gene [49] followed by labeling with biotin-16-dUTP as described in [48]. The probe for 35S rDNA was a clone that includes part of the 18S rDNA isolated from *Allium cernuum*, which was labeled with digoxigenin-11-dUTP as described in [48]. Sites of probe hybridization were detected using 20 μg mL^-1 ^fluorescein-conjugated anti-digoxigenin immunoglobulin (GE Healthcare, Chalfont St Giles, Buckinghamshire, UK) or 5 μg mL^-1 ^Cy3-conjugated avidin (Roche Pharmaceuticals, Lewes, East Sussex, UK) in 4 × SSC containing 0.2% (v/v) Tween 20 and 5% (w/v) bovine serum albumin. Chromosomes were counterstained with 2 μg mL^-1 ^DAPI (4',6-diamidino-2-phenylindole (Sigma-Aldrich Company Ltd. Dorset, UK) in 4 × SSC) and stabilized in Vectashield medium (Vector Laboratories Ltd., Peterborough, UK) prior to data acquisition using a Leica DMRA2 epifluorescent microscope fitted with an Orca ER camera and Open Lab software^® ^(Improvision, Coventry, UK). The images were adjusted with Adobe Photoshop^® ^version 7 and treated for color contrast and uniform brightness only. At least five mitotic cells per plant were scored with each probe used.

### DNA isolation, Southern blotting

Genomic DNA was extracted either from fresh leaves or leaves preserved in RNA*later *reagent (Applied Biosystems, Ambion, Warrington, UK), following the instructions of the manufacturer of RNA*later *or by a standard CTAB method described in [50] and modified in [51]. DNA concentration was estimated by two independent methods: (i) a CYBR green fluorescence method following a protocol at http://www.dnagenotek.com/); the green fluorescence was measured on a Rotor gene thermocycler (Corbett Research, Brisbane, Australia) as recommended; and (ii) ethidium bromide fluorescence measured after gel electrophoresis using phage lambda DNA as a standard. The estimates obtained from both methods were concordant. Integrity of DNA was checked on gels.

Southern blotting followed the protocol described in [51] using rDNA probes labeled with [α-^32^P]dCTP (Izotop, Budapest, Hungary). The ITS-1 probe was a *Bst*NI fragment from the cloned 18S-ITS-5.8S subregion of *T. mirus *rDNA (GenBank: AY458586). The 18S rDNA probe was a cloned 1.7-kb fragment of the tomato 18S rRNA gene [52], and the 26S rDNA probe was a 280-bp PCR product derived from the 3' end of the tobacco 26S rRNA gene [53]. Hybridization signals were visualized by phosphorimaging (Storm, Molecular Dynamics Sunnyvale, CA, USA).

For rDNA quantification, 17.5-200 ng of DNA were denatured in 0.2 M NaOH and loaded on a nylon membrane (Hybond XL, GE Healthcare, Little Chalfont, UK) using a slot blot apparatus (Schleicher & Schuell, Sigma-Aldrich, Dorset, UK). The membranes were hybridized with respective DNA probes in a Church Gilbert buffer [54]. Radioactivity in each band was counted using a rectangle integration method (ImageQuant software, GE Healthcare, Little Chalfont, UK). A standard curve was constructed using a diluted plasmid carrying the 18S rDNA insert [52]. The experiments were repeated three times and data averaged.

Statistical calculations were carried out using a chi-square function implemented in MicroSoft Excel.

## Results

### Plants and scheme of experiments

**Figure 1:**
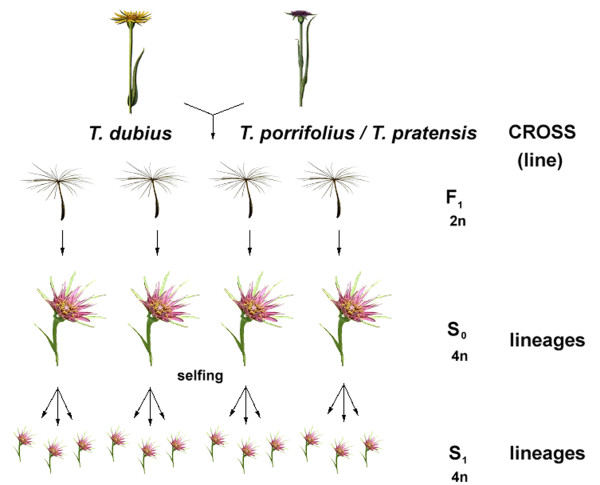
**Scheme of crossing strategies**. *Tragopogon dubius *and *T. porrifolius *(or *T. pratensis*) were used as either the maternal or paternal parent; each cross (line) gave rise to sterile diploid F_1 _progeny. To double chromosomes and restore fertility of these hybrids, seeds were treated with colchicine, obtaining fertile allotetraploid S_0 _plants. These individuals were selfed to produce lineages of fertile allotetraploid hybrids (generation S_1_, S_2_).

The generation of allotetraploid lines and phenotypic and karyological analysis were described elsewhere [47]. Briefly, the allotetraploids were derived from independent crosses (Figure 1) involving two or three different accessions of diploid *T. dubius*, *T. porrifolius*, and *T. pratensis *(Table 2). In this study, we used seven lines of synthetic *T. mirus*, four lines of synthetic *T. miscellus*, and two lines of diploid F_1 _hybrids. The parental origin for each line is given in Table 1. A "line" was defined as the progeny originating from a single cross (S_0_, S_1_, S_2_...) and maintained through selfing. "Lineages" were obtained by selfing of plants from the same cross but from different F_1 _seeds.

### Copy number estimates in diploid genome donors

Because estimates of rDNA amounts may vary among populations of diploid species, we determined gene copy number of diploid parents using slot blot hybridization. Two to six individuals from each population were analyzed. The DNA was hybridized on blots with the 18S rDNA probe and the amount of radioactivity estimated. An example of our hybridization analysis is shown in Additional file 1. It is evident that the strongest hybridization signals were obtained with DNA from *T. dubius *2613 and *T. porrifolius *2607; these were scored as "high-copy" accessions. On the other hand, *T. dubius *2615 and *T. porrifolius *2611 showed relatively weak hybridization signals and were scored as "low-copy" accessions. Variation within populations and among progeny was low (< 15%) or negligible. The copy number estimates for each population are given in Table 2.

### Southern blot hybridization in parental plants

**Figure 2:**
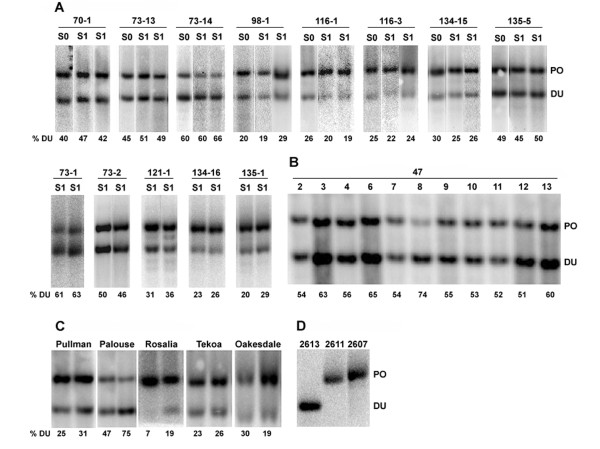
**rDNA repeat inheritance in *T. mirus *determined by Southern blot hybridization**. (A) Synthetic allotetraploid lines of *T. mirus*. (B) Diploid *T. porrifolius *x *T. dubius *hybrids. (C) Natural populations of *T. mirus*. (D) Profiles of parental diploids. Representative individuals are shown in the panels; the rest of the analysis expressed as a ratio of parental bands is shown in Figure 5A. Each S_0 _and S_1 _generation of synthetic allopolyploid (A) is encoded as follows: e.g. in 70-1, the first number is the line number, the second number indicates a particular lineage (Figure 1 and Table 1). The DNAs were digested with *Bst*NI and hybridized with the ITS-1 probe. The number below each lane indicates percentage of *T. dubius *units out of total rDNA. DU - *T. dubius *units; PO - *T. porrifolius *units.

**Figure 3:**
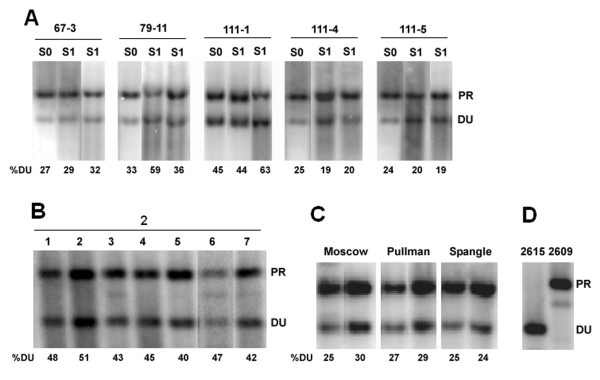
**rDNA repeat inheritance in *T. miscellus *determined by Southern blot hybridization**. (A) Synthetic allotetraploid lines of *T. miscellus*. (B) Diploid *T. pratensis *x *T. dubius *hybrids. (C) Natural populations of *T. miscellus*. (D) Profiles of parental diploids. Representative individuals are shown in the panels; the rest of the analysis expressed as a ratio of parental bands is shown in Figure 5B. The digests and conditions of the blot and labels are the same as in Figure 2. DU - *T. dubius *units; PR - *T. pratensis *units.

The ITS region was analyzed taking advantage of a diagnostic *Bst*NI restriction site polymorphism that distinguishes the diploid parents. In *T. dubius*, ITS-1 contains a *Bst*NI site close to the 5.8S gene while there is no *Bst*NI site in the ITS-1 of *T. porrifolius *and *T. pratensis *[45]. Consistent with previous results, the ITS-1 probe hybridized to the ~ 700-bp fragment in *T. pratensis *and *T. porrifolius *and to the ~ 500-bp fragment in *T. dubius *(Figures 2D, 3D).

The IGS region was analyzed using *Bst*YI and *Ssp*I restriction enzymes (Additional file 2). There are more *Bst*YI sites in the IGS of *T. porrifolius *than in *T. dubius*, based on the sequenced clones [GenBank: FN666261.1 and FN645941.1]. Consequently, the probe hybridized to low-molecular-weight fragments in *T. porrifolius *and to high-molecular-weight fragments in *T. dubius*.

### FISH analysis in parental plants

**Figure 4:**
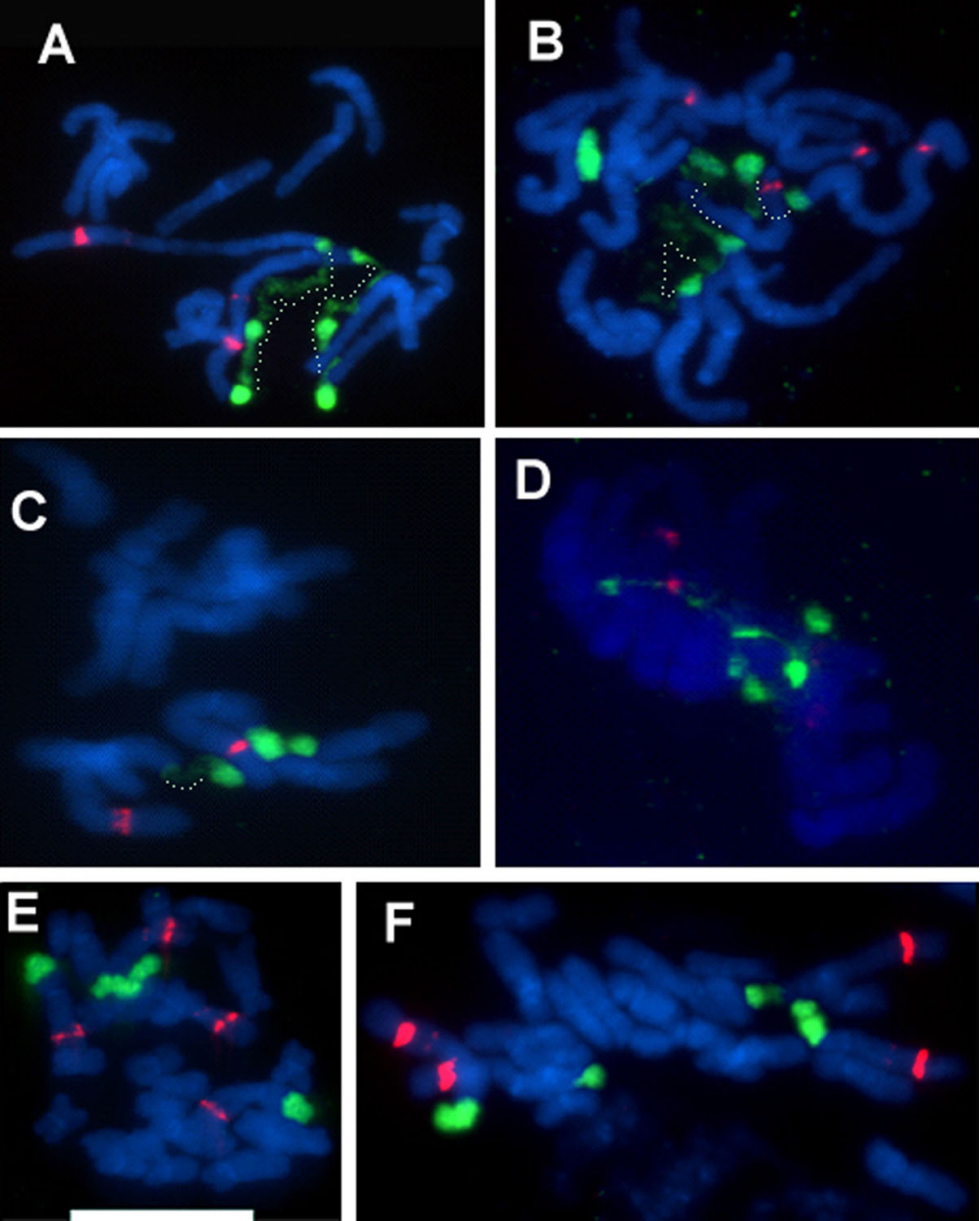
**FISH to metaphase spreads of parental diploid species (A-D) and synthetic *T. miscellus *lines (E-F)**. (A) *T. pratensis *2608. (B) *T. porrifolius *2607. (C) *T. dubius *2613. (D) *T. porrifolius *2611. (E) and (F) stand for the 111-1 and 111-4 synthetic individuals, respectively. Metaphases were hybridized with the 18S rDNA (painting 35S sites in green) and 5S rDNA (red fluorescence) probes. Note the discontinuous chromatin condensation along the loci. Regions of condensed and decondensed chromatin are interconnected with dotted lines in (A-C). Scale bar = 10 μm.

To determine the number and chromosomal position of rRNA genes, we carried out rDNA-FISH on metaphase chromosomes. In *T. dubius *(Figure 4C), there were two sites of probe hybridization for both 35S and 5S rDNA, located on the largest chromosome pair (A^du^; the nomenclature follows that of Pires *et al. *[55]). *T. pratensis *also has two sites of 35S rDNA (both decondensed) and one pair of 5S rDNA loci at metaphase (Figure 4A). Both 35S and 5S rDNA loci occur on chromosomes A^pr^. *T. porrifolius *has four 35S and four 5S rDNA sites at metaphase (Figures 4B, D). Chromosome A^po ^carries both 35S and 5S rDNA loci, at homeologous loci to the other diploids, but there are also 35S rDNA loci on the two homologs of chromosome D^po^, and 5S rDNA loci on both homologs of chromosome F^po^. The 5S signal on A^po ^was much weaker in accession 2611 (Figure 4D) than in accession 2607 (Figure 4B). The FISH results are consistent with previous cytogenetic analysis carried out in the same diploid populations, but with different individuals [55], indicating chromosomal stability of loci.

### Molecular and cytogenetic analysis in synthetic hybrids and allotetraploids

**Figure 5:**
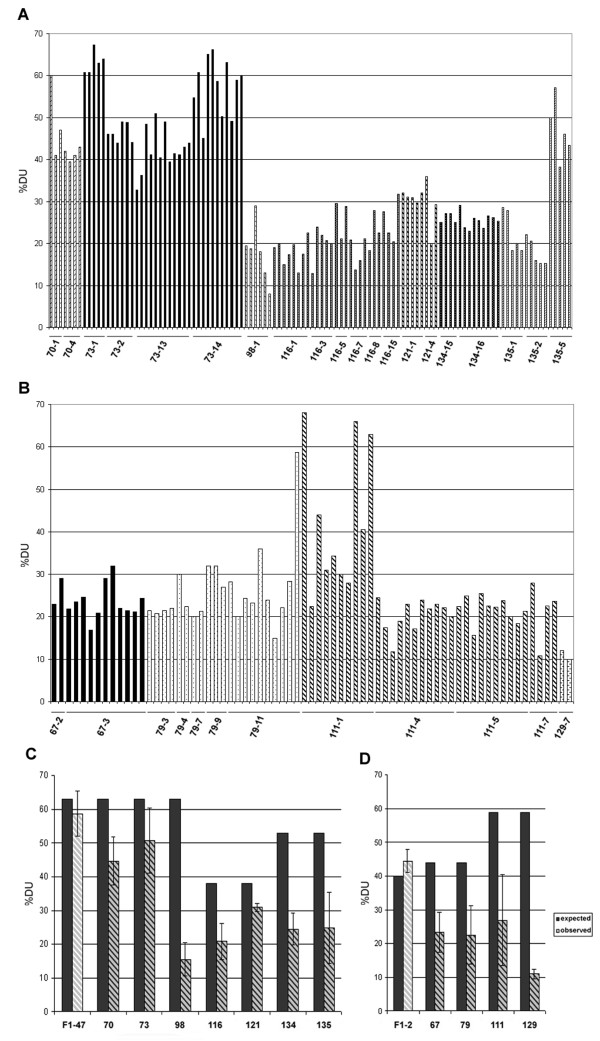
**Summary of rDNA repeat ratios analysis estimated for individual synthetic *T. mirus *(A, C) and *T. miscellus *(B, D) plants**. Homeolog gene ratios were determined from quantification of band signals after the Southern blot hybridization (Figures 2, 3). Each parental hybridization band was quantified using phosphorimaging, and gene ratios were expressed as a percentage of *T. dubius *units out of the total signal (A, B). The averaged values for each line are shown in graphs (C and D - shaded bars); for F_1 _diploid hybrids the shaded bars are drawn at low contrast. Filled bars represent expected gene ratios based on strict Mendelian inheritance of gene copy numbers. The interindividual variability within the line is expressed by standard deviation from the mean. Differences between expected and observed ratios were highly significant (P < 0.001, standard chi-square test) in all lines except the diploid F_1_-47 and F_1_-2 hybrids and line 121.

**Figure 6:**
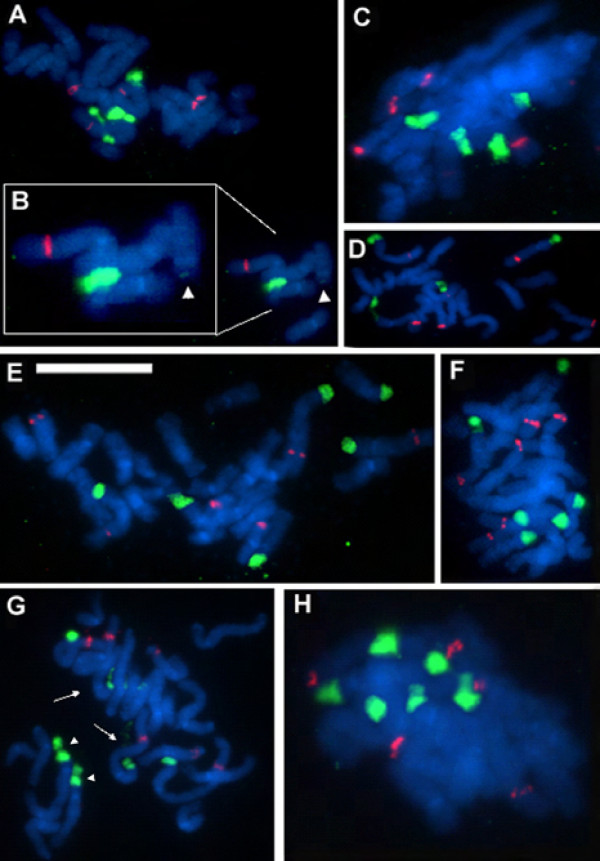
**FISH to metaphase spreads of synthetic *T. mirus***. The probe labels and scale bar are as in Figure 4. Individuals 73-14 (A, B), 70-4 (C), and 73-13 (D) had four strong plus 0-2 very weak signals (dependent on particular metaphase and sample). (B) is an expanded region of (A) showing the large locus of *T. dubius *origin and the minute D^po ^locus (arrowheads) left after the deletion of the majority of genes from the array. Lineages 98-1 (E), 134-15 (F), 116-1 (G), and 135-5 (H) showed regularly six 18S rDNA signals. The metaphase in (G) has largely decondensed A^du ^(arrows), partially decondensed D^po ^(arrowheads), and fully condensed A^po ^loci.

We carried out a population-level study of 35S rRNA gene copy number using Southern blot hybridization using the ITS-1 probe (Figures 2, 3). The ratios of gene units in each sample are shown in Figures 5A (*T. mirus*) and 5B (*T. miscellus*); the averaged values for each line are summarized in Figure 5C, D. FISH with the 18S rDNA probe was then carried out on selected individuals (Figures 4, 6 and Table 3). In the following sections, we describe the results of both types of analysis for each line derived from diploid parents with high, medium, or low copy numbers at the 35S rDNA loci (called high-, medium-, or low-copy) parents.

**Table 3:** Summary of cytogenetic analysis

*Species*	*^3^N*	*35S rDNA sites*	*5S rDNA sites*
Synthetic *T. mirus*^1^	17	4-6	6-7
Natural *T. mirus*^2^	11	6^4^	6-7
Synthetic *T. miscellus*^1^	2	4	4
Natural *T. miscellus*^2^	6	4	4

^1^this study.

^2 ^references [45,46,55].

^3^number of individuals analyzed by rDNA-FISH.

^4^some individuals from population Rosalia [45] had a nearly deleted locus on chromosome A^du ^inherited from *T. dubius *(Figure 2C).

### F_1 _diploid hybrids

We analyzed rRNA gene ratios using Southern blot hybridization with the ITS-1 probe against diploid F_1 _hybrid plants. In 11 F_1 _individuals (cross 47) from a single cross involving a "high-copy" *T. dubius *2613 paternal parent and a "low-copy" *T. porrifolius *2611 maternal parent (Figure 2B), the probe hybridized strongly to the *T. dubius*-origin rDNA units (DU) forming a lower molecular weight band, while the upper band of *T. porrifolius*-origin rDNA units (PO) was significantly weaker. Radioactivity scanning revealed that on average 60% of the total hybridization signal occurred in the lower molecular weight band, representing rDNA units of *T. dubius *origin. This value is close to the expected ratio considering the unit copy numbers in the diploid parents (Table 2 and Figure 5C, D). With the exception of one individual (47-8), plant to plant variation in gene ratios was low (Figure 2B). The second diploid hybrid (cross 2) examined was derived from a cross involving "low-copy" *T. dubius *2615 and "medium copy" *T. pratensis *2607 (Figure 3B). In this case, the upper molecular weight band of *T. pratensis-*origin rDNA units (PR) was stronger than the band inherited from *T. dubius*. Again, F_1 _hybrids generally showed the expected number of genes inherited from their parents (Figure 5C, D).

### Synthetic *T. mirus*

#### Line 73

This line was derived from a cross involving "low-copy" 2611 *T. porrifolius *(maternally inherited) and "high-copy" *T. dubius *2613 (paternally inherited). Thirty-seven individuals obtained from four lineages (73-1, 73-2, 73-13, and 73-14) were analyzed by Southern blot hybridization using the ITS-1 probe (examples are shown in Figure 2A). There was significant variation in rRNA gene ratios, and at least two distinct rDNA genotypes were distinguished: lineages 73-2 and 73-13 had nearly balanced DU/PO ratios, while lineages 73-1 and 73-14 had ratios skewed towards *T. dubius-*origin rDNA (61-75%) (Figures 5A and 5C). The 26S rDNA probe, which mapped polymorphisms in the intergenic spacer (IGS), showed similar results (Additional file 2). The homeolog gene ratios established in the S_1 _seem to be inherited among the S_2 _individuals (Additional file 3).

Representative individuals of both genotypes were analyzed by FISH (Figure 6A, B and 6D). The 18S rDNA probe hybridized strongly to terminal regions of both homeologs of chromosomes A^po ^and A^du^. However, there was no, or very little, hybridization signal on chromosome D^po ^(Figure 6B arrowheads), indicating locus loss or drastic elimination of units. This pattern appears to be typical for all S_1 _individuals and their progeny (Additional file 4). There were also quantitative differences in signal intensities. While lineages 73-2 and 73-13 had comparable sizes of all four 35S rDNA loci, lineages 73-1 and 73-14 had enlarged A^du ^loci. The number of 5S sites was additive (6 signals) in the S_1 _generation while one S_2 _individual gained a strong site likely on chromosome F (Additional file 4D). Thus, cytogenetic and molecular analysis both show considerable variability in rDNA locus sizes in these lineages.

#### Line 70

These allopolyploids were derived from "low-copy" *T. porrifolius *2611 (maternally inherited) and "high-copy" *T. dubius *2613 (paternally inherited), but different individuals were used as parents than in crosses 73 and 98. On the Southern blots, the ratios of parental bands were mostly balanced (Figure 2A). As in line 73, there were only four 35S rDNA signals (some with secondary constrictions) instead of the expected six, indicating locus loss (Figure 6C).

#### Line 98

Only a single line, 98-1, was recovered from this cross, originating from populations of "low-copy" *T. porrifolius *2611 (maternally inherited) and "high-copy" *T. dubius *2613 (paternally inherited). Different individuals were used as parents than in the previous two lines. On blots, the probe showed a much stronger hybridization to the band corresponding to the *T. porrifolius*-origin homeolog than to the homeolog of *T. dubius *origin (Figure 2A). There were six 35S and six 5S rDNA chromosomal sites, indicating an additive number of loci from that expected of the diploid parents (Figure 6E).

#### Line 116

In this cross we used "high-copy" *T. porrifolius *2607 as the maternal genome donor and "low-copy" *T. dubius *2615 as the paternal genome donor. All individuals from eight lineages showed relatively uniform Southern hybridization profiles, with band ratios highly skewed towards the *T. porrifolius*-origin units (Figures 2A, 5). FISH showed the expected number of 35S signals, with signals on chromosomes A^po^, D^po^, and A^du ^, although the signals on chromosome A^du ^(arrows) are smaller and highly decondensed (Figure 6G). On the other hand, the 35S signals on *T. porrifolius *chromosomes were strong, and NORs on D^po ^(arrowheads) showed only partial decondensation.

#### Line 121

This line originated from a cross involving "low-copy" *T. dubius *2615 as the maternal parent and a "high-copy" *T. porrifolius *2607 as a donor of the paternal genome. The gene ratio of all eight allotetraploid individuals was slightly shifted towards the *T. porrifolius *homeolog (Figure 5A). FISH was not conducted on this line.

#### Line 134

This line was derived from a "high-copy" *T. porrifolius *2607 (maternally inherited) and a "high-copy" *T. dubius *2613 (paternally inherited) and yielded two lineages (134-15 and 134-16). On the blots, individuals from both lines showed a relatively weak *T. dubius*-origin band and a strong *T. porrifolius*-origin band (Figures 2A, 5). At the cytogenetic level, the individuals inherited an additive number of 35S rDNA loci, four strong sites and two weaker ones, the latter likely of *T. dubius *origin, that are slightly remote from the bulk of the chromosomes due to secondary constriction (Figure 6F).

#### Line 135

This line originated from a cross reciprocal to that giving line 134 and comprised three lineages (1, 2, and 5). While both lineages 135-1 and 135-2 had gene ratios skewed towards *T. porrifolius*-origin units, the gene ratios were balanced in the 135-5 individuals (Figures 2A, 5). There was some variability among the 135-5 individuals (Figure 5A). However, the number of loci was additive in the individual examined using FISH (Figure 6H).

### Synthetic *T. miscellus*

#### Line 67

In this cross, a "high-copy" *T. pratensis *2609 was used as the maternal genome donor and "low-copy" *T. dubius *2616 as the paternal genome donor. There was little or no variation in the gene ratios among the individuals analyzed, and all individuals had reduced Southern hybridization signal against the *T. dubius*-origin units (Figure 3A).

#### Line 79

This cross is a reciprocal cross to that which generated line 67, and it yielded 5 lineages. There was some, albeit little, intralineage variability in band hybridization signal intensities. For example, the S_1 _individual in the middle lane (Figure 3A) had a stronger *T. dubius*-origin band compared to that from the other parent (*T. pratensis*) while most other members of this line had a dominant band of *T. pratensis *origin (Figure 5B).

#### Line 111

In this cross a "medium-copy" *T. pratensis *2608 (maternally inherited) and "high-copy" *T. dubius *2613 (paternally inherited) were used as genome donors. This combination of parents yielded the largest number of allotetraploid individuals (35) out of the *T. miscellus *crosses attempted. Lineages 111-4, 111-5, and 111-7 had relatively uniform profiles of Southern hybridization bands with ratios skewed away from the units of *T. dubius *origin (Figure 3A). Lineage 111-1 differed in having relatively large intralineage variability (Figure 5B). There were individuals with signals skewed towards the *T. pratensis *type as well as individuals with balanced gene ratios. FISH was carried out on randomly selected individuals of lineages 111-1 (Figure 4E) and (Figure 4F). Both plants retained an additive number of 35S and 5S rDNA loci from that expected of the diploid parents (i.e., four 35S and four 5S rDNA sites). However, plant 111-4 had two larger and two smaller 35S rDNA signals, all with secondary constrictions, while individual 111-1 had signals of comparable sizes on all four chromosomes.

#### Line 129

Line 129 resulted from a cross reciprocal to that which generated line 111. Only two allotetraploid individuals were recovered from this cross, and both had gene ratios highly skewed away from *T. dubius*-origin units (Figure 5B).

## Discussion

### rRNA gene copy number variation in synthetic *T. mirus *and *T. miscellus*

We analyzed the inheritance of rRNA genes in seven lines of synthetic *T. mirus *and four lines of synthetic *T. miscellus*. In synthetic *T. mirus*, 32 individuals (29%) exhibited balanced rDNA genotypes, 69 individuals (63%) showed more 35S rDNA of *T. porrifolius *origin than expected, and only 9 plants (8%) had more *T. dubius-*origin rDNA (Table 4). In synthetic *T. miscellus*, three individuals (4%) had balanced gene ratios while 65 individuals (92%) inherited more *T. pratensis*-origin units than expected. The genetic variation in copy ratios among the progeny of a single cross ranged from low or negligible (5%, line 134) to high (40%, line 111) (Figure 5C). Some crosses involving the same parental accessions (lines 70, 73, and 98) gave rise to individuals with expected ratios of parental 35S rDNA units considering the copy numbers in the diploid parents, while others had rDNA genotypes balanced or skewed towards units derived from either of the diploid parents.

**Table 4:** Comparison of rDNA ratios in synthetic and natural populations of allotetraploids

*Species*	*Genotype*	*Gene ratio*, *Du:Po/Pr*	*^1^N*	*% of individuals*
Synthetic *T. mirus*	high Du	> 60%	9	8
	balanced	40-60%	32	29
	high Po	> 60%	69	63
	total		110	
				
Natural *T. mirus*	high Du	> 60%	0	0
	balanced	40-60%	20	29
	high Po	> 60%	48	71
	total		68	
				
Synthetic *T. miscellus*	high Du	> 60%	3	4
	balanced	40-60%	3	4
	high Pr	> 60%	65	92
	total		71	
				
Natural *T. miscellus*	high Du	> 60%	0	0
	balanced	40-60%	0	0
	high Pr	> 60%	31	100
	total		31	

^1^N - number of individuals. The data were obtained from the S_1 _generation of synthetic lines (Table 1); natural populations were analyzed in [45,60].

### Sources of rDNA copy number variability in allotetraploids

#### (i) Contribution of natural variation in parental accessions

There may be up to two-fold variation in 35S rDNA copy number between different accessions of the same *Tragopogon *species. The differences in copy number were larger between populations of the same species than between species. These data indicate that shifts in rDNA array sizes occur at the lineage level. Similar levels of interpopulation variability were reported among *Arabidopsis *accessions [56]. As a consequence, hybridizing species may inherit a variable number of rRNA genes depending on the parental populations involved. This hypothesis has been tested in F_1 _diploid hybrids. For example, a "low-copy" *T. dubius *accession 2615 combined with a "high-copy" *T. porrifolius *2607 should generate skewed 1:2 DU/PO ratios in a hybrid (lines 116 and 121, Figure 5C). Conversely, a "high-copy" *T. dubius *2613 combined with a "low-copy" *T. porrifolius *2611 should result in a 2:1 DU/PO gene ratio. Indeed, the analysis of F_1 _diploid hybrids confirmed the unequal gene dosage inherited from both parents (Figures 2, 3, and 5). However, many allopolyploid lines involving a "high-copy" *T. dubius *parent showed far fewer copies of this parental type than expected, suggesting that standing variation in diploids does not account for all observed rDNA imbalances in the derived polyploids.

Another source of "inherited variation" may stem from heterozygosity in locus sizes in the parents. For example, in *Streptocarpus *a major rDNA locus occurs in a hemizygous condition, accounting for gene copy number variation in derived hybrids [57]. This sort of heterozygosity would be evident if there were a very low-copy array and a very high-copy array in parents. However, FISH analysis revealed no indication of rDNA heterozygosity in the diploid *Tragopogon *individuals investigated, a situation which might be expected for species that are largely selfing [58,59], and in plants that were derived from inbred lines propagated in a greenhouse (at least one generation). In estimating rDNA locus sizes, and hence relative copy numbers at individual loci, FISH may be influenced by the condensation state of the chromatin. However, we examined many cells in making our assessments and did not observe heterozygozity even in the most condensed metaphases. Furthermore, our data revealed clear associations between the size of the FISH signal and the number of rRNA gene copies estimated by Southern hybridization. Most F_1 _diploid hybrids showed gene copy number ratios that were close to expectation. However, one F_1 _individual (47-8) resulting from the cross *T. dubius *2613 × *T. porrifolius *2611 showed altered homeolog gene ratios from Mendelian expectation (Figure 2B). Further, segregation of IGS sequence polymorphisms upon selfing of parental diploids was noted in some cases (Hana Malinska - unpublished). Whether these low-frequency events reflect heterozygosity in locus sizes, gametic variation or postzygotic changes is currently unknown.

In short, the genetic variation among the parents contributed some, but certainly not a major portion of the rDNA variability seen in the allotetraploids.

#### (ii) Contribution of allopolyploidy-related factors to rDNA variability

We observed deviations from expected gene ratios in most of the synthetic tetraploid individuals we examined (Figures 5C, D). The Southern blot and FISH data further show that this was mainly caused by underrepresentation of *T. dubius*-origin units. Directionality of the change is not influenced by the partner genome being either *T. pratensis *or *T. porrifolius *in origin, as similar patterns occurred in both synthetic *T. mirus *and *T. miscellus*. However, the extent and frequency of these changes differed among the lines of the same species. For example, the expected 2:1 DU/PO ratio of units has been reversed into a 1:4 ratio in line 98 of synthetic *T. mirus*, whereas in other lines the ratios changed relatively little (line 121). In general, deviation from repeat additivity occurred more frequently in lines resulting from crosses involving *T. dubius *2613 as a parent than any other accession (Figure 5). The contribution of parental cytoplasm to gene imbalances could be assessed from the analysis of reciprocally formed individuals. The average DU/PO gene ratios in reciprocally formed lines (134 and 135) of *T. mirus *were comparable (Figure 5C) although variation was higher in line 135 that inherited *T. dubius *2613 as a mother genome donor. However, relatively few individuals (28) were sampled to allow firm conclusion on this topic. Variation within lines was generally lower than that between the lines. Nevertheless, members of lineage 111-1 (synthetic *T. miscellus*) did show much variation (Figure 5B), and most segregating progeny differed from the parental genotype.

Repeat number variation was reflected by differences in 35S rDNA locus sizes. For example, line 116 of synthetic *T. mirus*, which has only 19% rDNA of *T. dubius *origin, showed small FISH signals on both A^du ^homologs in contrast to signals on A^po ^and D^po^; their small sizes are indicative of loss in rDNA repeats at this locus (Figure 6G). However, the high level of decondensation occurring at one or both A^du ^sites suggests a high level of transcriptional activity at the preceding interphase. Indeed, RT-PCR experiments confirmed strong expression dominance of *T. dubius *loci in this (Additional file 5) and other lines of synthetic *T. mirus *and *T. miscellus *(Hana Malinska - unpublished). High transcriptional activity from an rDNA locus with reduced rRNA gene copy numbers has also been reported previously for natural *T. mirus *and *T. miscellus *individuals [60]. Similarly, FISH revealed that lineage 111-4 of *T. miscellus *had two small and two large rDNA loci (Figure 4F) while a sister lineage, 111-1, had four large sites at both rDNA loci on chromosomes A^pr ^and A^du ^(Figure 4E). Line 111-1 also had more *T. dubius*-origin gene copies than individual 111-4 (Figure 3A). Thus, FISH analysis confirmed that the shifts in rRNA gene ratios were likely caused by contractions of repeats on the A^du ^locus. Synthetic *T. mirus *lineage 73-14 is a notable exception in having an extremely large A^du ^locus (Figures 6A, B and Additional files 4A, B, C). This may have been caused by amplification of repeats within the locus, but the plants also have a smaller locus on the other A^du ^homolog, potentially indicating translocation of repeats between homologs, perhaps as a consequence of unequal recombination at meiosis.

### Mechanisms of rDNA rearrangements

Meiotic aberrations have been implicated in rRNA gene imbalances in natural populations of *Tragopogon *(e.g., [46]). Indeed, meiotic analysis of eight S_1 _plants (same material as used in this study) revealed a number of abnormalities, including multivalent formation, lagging chromosomes, and aneuploidy [47]. Chromosomes bearing rDNA appeared to be frequently involved in the formation of bridge complexes. Interestingly, lines involving *T. dubius *2613 (70 and 98) showed higher frequencies of both meiotic irregularities and rDNA rearrangements than lines derived from other accessions. There seems to be a good correlation between meiotic pairing abnormalities and frequency of rDNA changes in *Tragopogon*. Ribosomal RNA gene copy number losses may occur through unequal recombination (perhaps also recombination between non-homologous or homologous chromosomes).

Shifts in gene ratios were also observed in some S_0 _plants (i.e., the premeiotic synthetic allotetraploids), arguing that meiotic irregularities may not be the only mechanism responsible for rDNA rearrangements, but mechanisms acting during or immediately after genome doubling could also be involved. Such mechanisms include recombination between rDNA loci (at homologous loci or otherwise), aneuploidy, and chromosome loss in somatic cells, the latter two having been reported in interspecific *Arabidopsis *hybrids and allopolyploids [61]. Reciprocal aneuploidy (loss compensated by gain of another chromosome of the complement) might explain the occurrence of four major 35S rDNA sites instead of six in *T. mirus *synthetic lines 70 and 73 (Figure 6). However, most synthetic polyploid individuals had the expected number of rDNA loci, and significantly altered gene ratios are likely arising through rearrangements targeted at the locus itself. In yeast, recombination between sister chromatids of the same chromosomes were shown to be a major source of array contractions and expansions [62,63]. A similar mitotic driven mechanism seems to be responsible for changes in copy number during development of *Vicia faba *[17] and in flax genotrophs [64]. Occasionally prolonged treatment with mitotic inhibitors causes the sister chromatids to separate but not to segregate as the cell proceeds towards anaphase. In one synthetic *T. mirus *cell (Additional file 4E) we observed that the chromatids were held together only at the rDNA loci, perhaps indicating unresolved recombination sites with rDNA, as can occur at the anaphase checkpoint in yeast [65,66]. Mitotic problems may underlie the high mortality of S_0 _generation plants in which > 50% of individuals did not survive or were sterile [47].

We have recently proposed that the nucleolus could be a site of inter- and intralocus recombination [67]. This hypothesis is supported by observation of increased homologous pairing at NORs in interphase of *Arabidopsis *[68]. Perhaps the decondensed chromatin of highly active genes promotes genetic recombination during interphase, resulting in the contraction/expansion of arrays. Another possibility is that subrepeated regions of the IGS may stimulate recombinogenic activity of units [17]. The IGS subrepeats in the sequenced *T. dubius *unit are longer and more homogeneous than those present in the *T. porrifolius *unit (Additional file 6). *T. dubius *arrays displayed greater level of variation than the *T. porrifolius *arrays in the allopolyploid lines.

### Comparison of natural and synthetic populations of allotetraploids

*T. mirus *and *T. miscellus *in the wild formed repeatedly within the last century and hence represent a unique system for studying the early stages of genome evolution following interspecific hybridization and genome duplication. In some areas, the progenitor diploids still occur along with expanding populations of the allotetraploids, and the polyploids at those locations likely represent descendents of the nearby diploid populations [69]. This scenario would apply, for example, to the *T. mirus *collections from Pullman, Washington. The collected *T. dubius *individuals from Pullman (2613) have nearly two-fold higher rDNA copy number compared to *T. porrifolius *(2611) at the same location. Yet, in both natural populations of *T. mirus *allotetraploids sampled, the *T. dubius *rDNA represents only 20-25% of the total rDNA [45] perhaps suggesting that ~75% of A^du ^repeats have been lost in the approximately 30-40 generations since the polyploids (which are biennials) formed at these locations. We were surprised to discover that one (98) out of three lines of *T. mirus *synthesized from Pullman parents showed a genotype that closely resembled that of both natural populations. The gene imbalances were, however, less pronounced in the other two lines (70, 73). In line 73 some individuals even had more *T. dubius *units, indicating that array size could be both maintained and altered during allopolyploidy. Interestingly, one natural population of *T. mirus *(from Palouse, Washington) also contained individuals with ratios skewed away from *T. porrifolius *(Figure 2 and Table 4). These examples illustrate that, as in other allopolyploid species [24,25], concerted evolution may occur bidirectionally in *Tragopogon *despite the prevalent trend towards contraction of *T. dubius *arrays in most plants and populations. In contrast to other systems [24,28,40,70,71], we have no molecular evidence for interlocus recombination of rDNA in either natural or synthetic populations of *Tragopogon *although sequence analysis of ITS clones has not been conducted in synthetic material.

Natural populations of allotetraploids of independent origin differ morphologically, biochemically, and genetically [39] although these allotetraploids apparently originated from a relatively narrow genetic pool of parental populations [69]. One explanation for interpopulational diversity in the allopolyploids is that genetic variation was triggered during the early generations post-allopolyploidization. This hypothesis is supported by an observation that the same parental accessions may give rise to lineages with differing rDNA genotypes. *Brassica napus *allotetraploids appear to share many features with the *Tragopogon *system, including homeolog pairing [72,73] and preliminary data that indicate that some rDNA loci may be lost in early generations (Ales Kovarik - unpublished). In contrast to rDNAs, low-copy, protein-coding sequences do not seem to be markedly altered in the early generations of the synthetic lines of *T. mirus *and *T. miscellus *[74]. Perhaps rearrangements of low-copy genes lag behind changes in the highly repeated fraction of the genome.

It is assumed that, as time passes, homogenization processes such as unequal crossing over continue to gradually replace parental rDNA arrays with novel allopolyploid species arrays. However, the time factor does not seem to be the only player. For example, while most Old World *Tragopogon *allotetraploids (assumed to be of ancient origin) homogenized ITS nearly to completion another old allotetraploid, *Tragopogon castellanus*, retained equivalent amounts of both parental ITS types [75]. Similarly, in rice [26] and *Glycine *[25] most, but not all, populations homogenized parental rDNAs. We envisage that the extent and tempo of rDNA homogenization in older allopolyploids is largely influenced by genetic and epigenetic changes in the early generations of allopolyploids. The fact that some rDNA genotypes seen in 80-year-old allopolyploids are already evident in the first generation of synthetic lines supports this hypothesis. However, this does not exclude the possibility that other changes in rDNA loci can occur gradually and stochastically over extended periods of time.

## Conclusions

We observed similar reductions of homeologous rRNA gene copies in both synthetic and natural, 80-year-old populations of *Tragopogon *allopolyploids, indicating that some aspects of genome evolution might be repeatable. The biological significance of gene losses as well as their potential adaptive significance is unclear. Uniparental deletions (partial or complete) would not affect fitness because there is a large excess of genes in the partner genome. One possibility is that intralocus rearrangements (translocation, deletions, amplification) preclude interlocus homogenization in older allopolyploids. The latter process is frequently associated with reduction of loci and repeats [67]. It is therefore possible that gene elimination/shrinkage of arrays serves as an alternative regulatory mechanism to epigenetic silencing, reducing the number of functional genes in a cell. Recently, Hawkins et al. [76] proposed that DNA loss may counterbalance genome expansion through retrotransposon proliferation. Perhaps rRNA gene elimination may reflect general tandem repeat instability in the allopolyploid nucleus. Retroelement activity in *Tragopogon *populations remains to be analyzed. Finally, rDNA arrays were recently shown to influence gene expression at ectopic positions in *Drosophila *[77], and locus size could potentially serve as an epigenetic regulator harmonizing the expression of subgenomes.

## List of abbreviations

ITS: internal transcribed spacer; IGS: intergenic spacer; FISH: fluorescent *in situ *hybridization; NOR: nucleolar organizer region; Organisms: *T. dubius*: *Tragopogon dubius*; *T. mirus*: *Tragopogon mirus*; *T. miscellus*: *Tragopogon miscellus*; *T. porrifolius*: *Tragopogon porrifolius*; *T. pratensis*: *Tragopogon pratensis*

## Authors' contributions

HM carried out most of the molecular biology and cytogenetic experiments. AK, DS, PS, and AL designed the study. AK wrote and drafted the paper. RM participated in the DNA analysis. AL carried out some FISH experiments. JT made the crosses and prepared synthetic lines. All authors read and approved the final manuscript.

## Supplementary Material

Additional file 1 **Slot blot quantification of gene copies in parental diploids**. The DNA amounts are indicated above each lane. The blot was hybridized with the ^32^P - labeled 18S rDNA probe. Experiments I and II were carried out in this study; experiment III is from [45]. Click here for file

Additional file 2 **Analysis of intergenic rDNA spacer polymorphisms in DNA of synthetic *T. mirus *(S_1 _generation)**. Genomic DNA was digested with *Bst*YI and *Ssp*I restriction enzymes. Southern blot hybridization was carried out using the 26S rDNA probe. Click here for file

Additional file 3 **Southern blot analysis of the S_2 _generation of synthetic *T. mirus***. Individuals were the progenies of three lineages from line 73. Click here for file

Additional file 4 **FISH analysis of the S_2 _generation of synthetic *T. mirus***. The same plants as in Additional file 3 were analyzed. Most metaphases displayed aneuploid karyotypes (23 chromosomes). Arrowheads in (C, F) indicate a minute D^po ^locus left after the deletion of the majority of genes. Note fusion of subtelomeric NORs at the chromatids (arrowheads, E) and considerable variability in condensation of rDNA chromatin among sister plants (A-C). The following individuals are shown: (A) - 73-14-6A, (B) - 73-14-6B, (C) - 73-14-6C, (D, E) - 73-1-3 D, (F) - 73-2-8B. Click here for file

Additional file 5 **Expression analysis of rDNA in synthetic *T. mirus *(line 116)**. RNA isolation and RT-CAPS assay were carried out as described in [60]. Note typical inverse correlation between gene copy number (grey bars) and their expression (black bars). Click here for file

Additional file 6 **Analysis of IGS subrepeats in *T. dubius *2613 and *T. porrifolius *2611**. We used a dot plot alignment tool at http://www.vivo.colostate.edu/molkit/dnadot/, self (x-axis) to self (y-axis) alignment (Window size: 9. Mismatch limit: 0). The IGSs were amplified using primers designed to conserved regions in 26S rDNA and 18S rDNA [10]. Briefly, the ~3.5-kb PCR products obtained were cloned into pSC-B-amp/kan vector using StrataClone Blunt PCR Cloning Kit (Stratagene, La Jolla, CA, USA). Clones bearing inserts of expected lengths were initially sequenced from both ends using universal M13 reverse and T7 primers. To obtain full-length sequence, the IGS-specific primers were designed based on the partial sequence. Five new primers were needed to cover the whole IGS region. Click here for file
