# Supplementary figures and images for: Similar patterns of rDNA evolution in synthetic and recently formed natural populations of Tragopogon (Asteraceae) allotetraploids
Source: BMC Evol Biol. 2010 Sep 22;10:291. doi: 10.1186/1471-2148-10-291 (PMC2955031; doi:10.1186/1471-2148-10-291)

### Experiment I

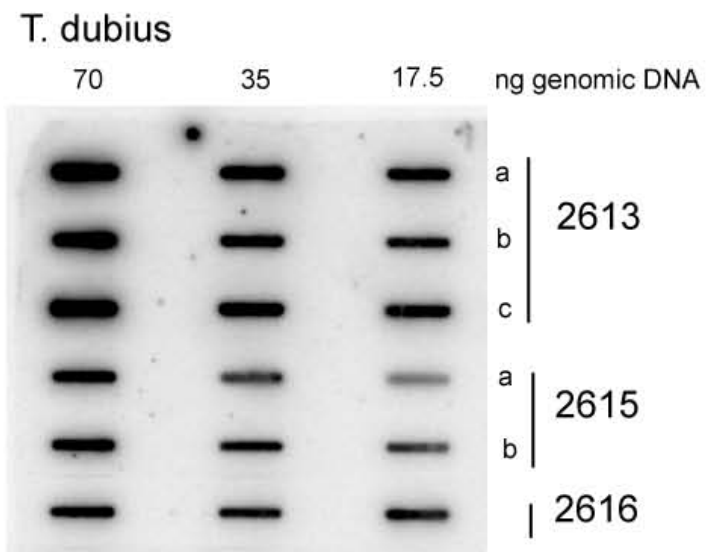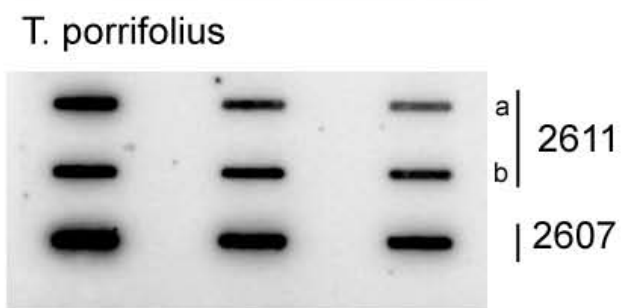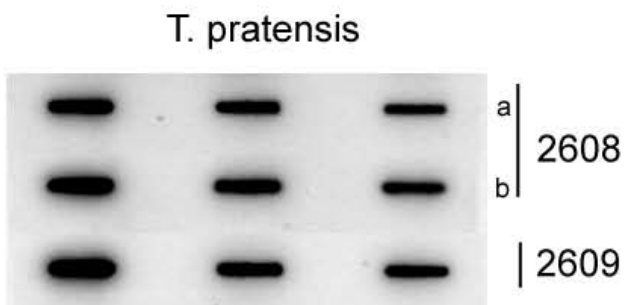

### Experiment II

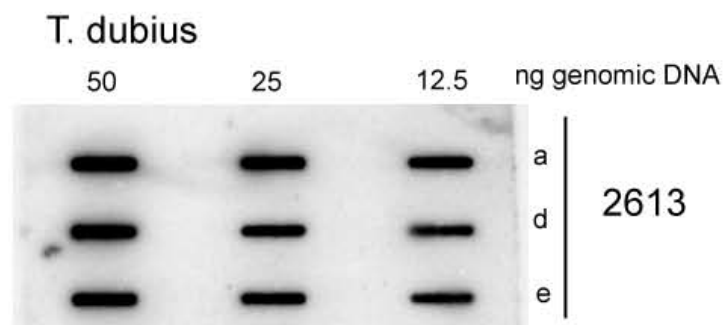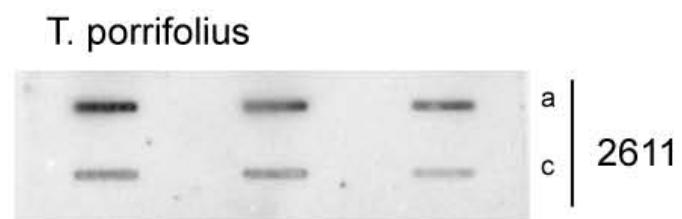

### Experiment III

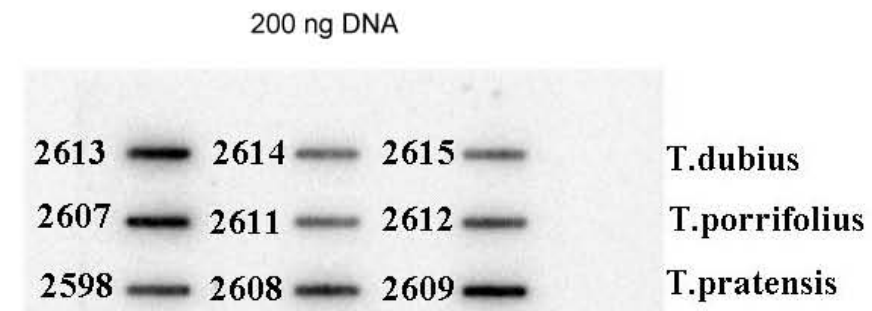

Supplement: Additional file 1 — Slot blot quantification of gene copies in parental diploids. The DNA amounts are indicated above each lane. The blot was hybridized with the 32P - labeled 18S rDNA probe. Experiments I and II were carried out in this study; experiment III is from [45]. [file 1471-2148-10-291-S1.PDF]

*T. porrifolius* 2611

*T. dubius* 2613

73-1

73-2

73-13

73-14

6

7

3

5

3

12

7

9

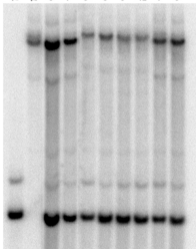

DU (7kb)

DU (5kb)

PO (2kb)

PO (1kb)

47

48

33

32

28

30

47

48

%DU

Supplement: Additional file 2 — Analysis of intergenic rDNA spacer polymorphisms in DNA of synthetic T. mirus (S1 generation). Genomic DNA was digested with BstYI and SspI restriction enzymes. Southern blot hybridization was carried out using the 26S rDNA probe. [file 1471-2148-10-291-S2.PDF]

***S<sub>2</sub> progeny of synthetic tetraploid lines 73***

**73-1**

**73-2**

**73-14**

**3B**

**3C**

**3D**

**3E**

**8A**

**8B**

**8C**

**6A**

**6B**

**6C**

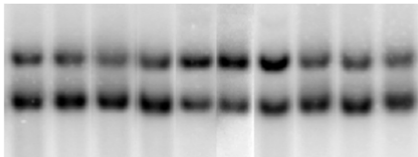

**PO**

**DU**

**%DU**

**60**

**63**

**63**

**62**

**46**

**41**

**44**

**59**

**61**

**61**

Supplement: Additional file 3 — Southern blot analysis of the S2 generation of synthetic T. mirus. Individuals were the progenies of three lineages from line 73. [file 1471-2148-10-291-S3.PDF]

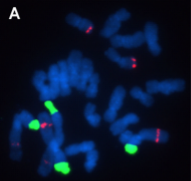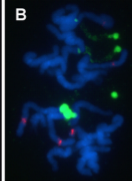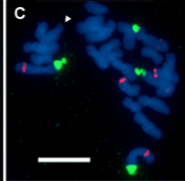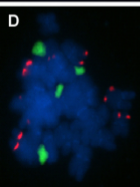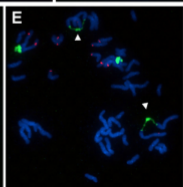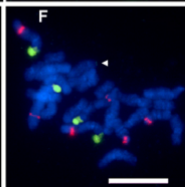

Supplement: Additional file 4 — FISH analysis of the S2 generation of synthetic T. mirus. The same plants as in Additional file 3 were analyzed. Most metaphases displayed aneuploid karyotypes (23 chromosomes). Arrowheads in (C, F) indicate a minute Dpo locus left after the deletion of the majority of genes. Note fusion of subtelomeric NORs at the chromatids (arrowheads, E) and considerable variability in condensation of rDNA chromatin among sister plants (A-C). The following individuals are shown: (A) - 73-14-6A, (B) - 73-14-6B, (C) - 73-14-6C, (D, E) - 73-1-3 D, (F) - 73-2-8B. [file 1471-2148-10-291-S4.PDF]

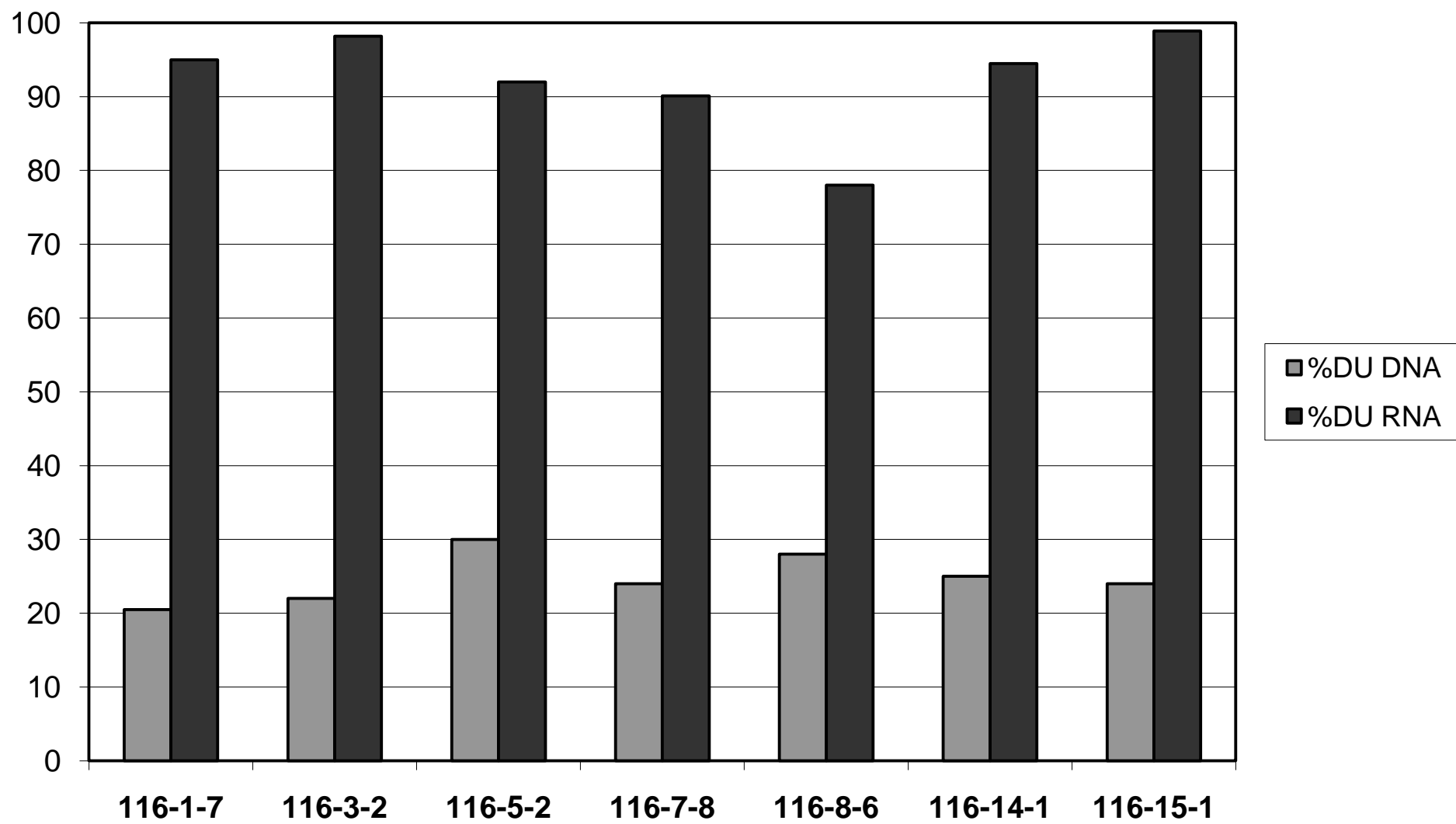

Supplement: Additional file 5 — Expression analysis of rDNA in synthetic T. mirus (line 116). RNA isolation and RT-CAPS assay were carried out as described in [60]. Note typical inverse correlation between gene copy number (grey bars) and their expression (black bars). [file 1471-2148-10-291-S5.PDF]

*T. porrifolius*

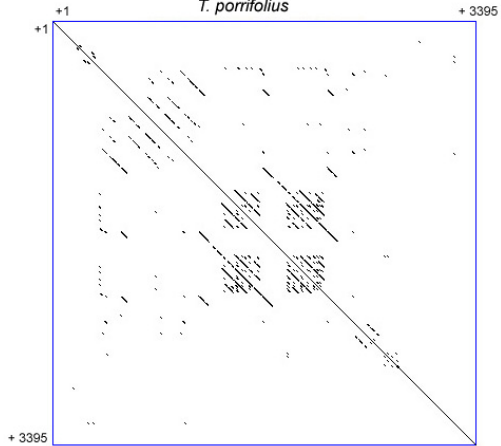

*T. dubius*

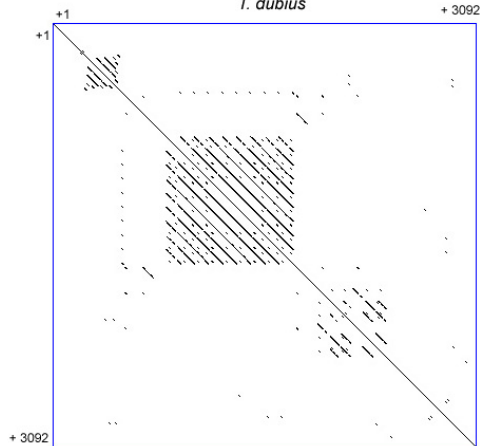

Supplement: Additional file 6 — Analysis of IGS subrepeats in T. dubius 2613 and T. porrifolius 2611. We used a dot plot alignment tool at http://www.vivo.colostate.edu/molkit/dnadot/, self (x-axis) to self (y-axis) alignment (Window size: 9. Mismatch limit: 0). The IGSs were amplified using primers designed to conserved regions in 26S rDNA and 18S rDNA [10]. Briefly, the ~3.5-kb PCR products obtained were cloned into pSC-B-amp/kan vector using StrataClone Blunt PCR Cloning Kit (Stratagene, La Jolla, CA, USA). Clones bearing inserts of expected lengths were initially sequenced from both ends using universal M13 reverse and T7 primers. To obtain full-length sequence, the IGS-specific primers were designed based on the partial sequence. Five new primers were needed to cover the whole IGS region. [file 1471-2148-10-291-S6.PDF]
